# Chaga mushroom extract suppresses oral cancer cell growth via inhibition of energy metabolism

**DOI:** 10.1038/s41598-024-61125-z

**Published:** 2024-05-09

**Authors:** Donghyeon Yeo, Yeo Gyun Yun, Seong-Jin Shin, Khandmaa Dashnyam, Anand Khurelbaatar, Jun Hee Lee, Hae-Won Kim

**Affiliations:** 1 Institute of Tissue Regeneration Engineering (ITREN), Dankook University, 119, Dandae-ro, Cheonan, 31116 Republic of Korea; 2 Department of Nanobiomedical Science and BK21 Four NBM Global Research Center for Regenerative Medicine, Dankook University, Cheonan, 31116 Republic of Korea; 3 Mechanobiology Dental Medicine Research Center, Dankook University, Cheonan, 31116 Republic of Korea; 4 Drug Research Institute, Mongolian University of Pharmaceutical Science, Ulaanbaatar, 18130 Mongolia; 5 Department of Biomaterials Science, College of Dentistry, Dankook University, Cheonan, 31116 Republic of Korea; 6 UCL Eastman-Korea Dental Medicine Innovation Centre, Dankook University, Cheonan, 31116 Republic of Korea

**Keywords:** Chaga mushroom extract, Oral cancer cell, Cancer progression, Glycolysis, Mitochondrial respiration, Cancer metabolism, Oral cancer, Autophagy, Cell death, Cell growth, Cancer, Cell biology

## Abstract

Oral cancer stands as a prevalent maligancy worldwide; however, its therapeutic potential is limited by undesired effects and complications. As a medicinal edible fungus, Chaga mushroom (*Inonotus obliquus*) exhibits anticancer effects across diverse cancers. Yet, the precise mechanisms underlying its efficacy remain unclear. We explored the detailed mechanisms underlying the anticancer action of Chaga mushroom extract in oral cancer cells (HSC-4). Following treatment with Chaga mushroom extracts, we analyzed cell viability, proliferation capacity, glycolysis, mitochondrial respiration, and apoptosis. Our findings revealed that the extract reduced cell viability and proliferation of HSC-4 cells while arresting their cell cycle via suppression of STAT3 activity. Regarding energy metabolism, Chaga mushroom extract inhibited glycolysis and mitochondrial membrane potential in HSC-4 cells, thereby triggering autophagy-mediated apoptotic cell death through activation of the p38 MAPK and NF-κB signaling pathways. Our results indicate that Chaga mushroom extract impedes oral cancer cell progression, by inhibiting cell cycle and proliferation, suppressing cancer cell energy metabolism, and promoting autophagy-mediated apoptotic cell death. These findings suggest that this extract is a promising supplementary medicine for the treatment of patients with oral cancer.

## Introduction

Oral cancer is one of the most common cancers worldwide, with almost 377,000 new diagnoses and 177,000 deaths estimated in 2020^1^. Numerous dangerous elements have been associated with oral cancer, such as tobacco use, alcohol exposure, and human papillomavirus infection^2^. Oral cancer causes changes in facial appearance, speech dysfunction, chronic pain, and dysphagia, leading to social isolation and psychological distress. Primary treatment options for oral cancer include surgery, radiation therapy, and chemotherapy either individually or in combination^3^. Nevertheless, treatment effectiveness is restricted by the potential for unwanted effects and complications, such as injury to healthy tissues, impaired speech and swallowing, and decreased quality of life^4,5^.

Edible medicinal mushrooms are a powerful source of bioactive molecules for the treatment of several tumor types. Chaga mushrooms (*Inonotus obliquus*) have various pharmacological effects on human health, including antitumor, anti-inflammatory, antioxidant, antimicrobial, and antiparasitic effects^6^. Chaga mushroom extract also has antitumor effects on several cancer types, such as human lung adenocarcinoma, hepatocellular carcinoma, and breast cancer^6–8^ as it contains bioactive compounds such as melatonin, polysaccharides, and triterpenoids that exhibit antitumor properties^6,9,10^. However, the underlying mechanism of the antitumor effects remains unknown.

Proliferating cancer cells have metabolic demands that differ from those of most differentiated cells^11^ in that cancer cells undergo glucose metabolism reprogramming, characterized by increased glucose uptake, high rates of glycolysis, and altered mitochondrial function. This metabolic phenomenon is commonly referred to as aerobic glycolysis, or the Warburg effect^12–14^. The metabolic adaptations exhibited by cancer cells enable them to acquire the energy and foundational components required for rapid proliferation and growth^15–17^. Therapeutic strategies aim to undermine these unique metabolic pathways by targeting cancer cell metabolism and exploiting their metabolic vulnerabilities. To effectively hinder the growth of cancer cells, it may be imperative to obstruct specific enzymes involved in glycolysis, target nutrient transporters, or modulate signaling pathways that regulate metabolism^18^. Thus, understanding and controlling the metabolic processes in cancer cells is a promising opportunity for the development of innovative and efficacious cancer treatments.

The aim of this study was to investigate whether Chaga mushroom extract affects the growth and metabolism of oral cancer cells. Alterations in cellular metabolism—including glycolysis and mitochondrial respiration—by treatment with Chaga mushroom extract are involved in oral cancer cell behavior, such as autophagy, cell cycle, and apoptosis. In particular, this study reports the inhibitory effect of this extract on oral cancer cells by regulating several cell-signaling pathways, including adenosine monophosphate-activated kinase (AMPK), signal transducer and activator of transcription 3 (STAT3), p38 mitogen-activated protein kinase (MAPK), and nuclear factor kappa-light-chain-enhancer of activated B cells (NF-κB) pathways.

## Results

### Chaga mushroom extract inhibits cell survival and proliferation in HSC-4 oral cancer cells

**Figure 1:**
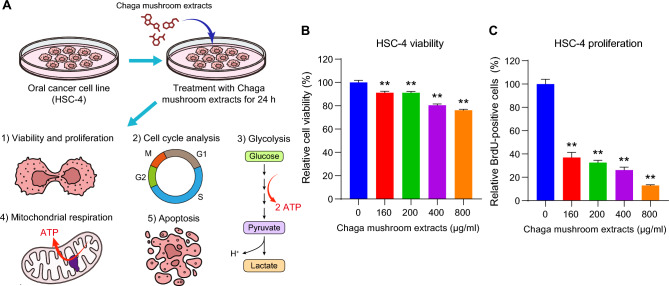
Chaga mushroom extract decreases the viability and proliferation of the oral cancer cell line HSC-4. (**A**) Graphical illustration of the experimental design used to analyze the viability and proliferation, cell cycle, glycolysis, mitochondrial respiration, and apoptosis of HSC-4 cells after treatment. (**B**) Viability of HSC-4 cells after treatment (0, 160, 200, 400, and 800 μg/mL). (**C**) Proliferation of HSC-4 cells after treatment (0, 160, 200, 400, and 800 μg/mL). Values represent the mean ± SEM. **p < 0.01 vs. untreated HSC-4 cells.

**Figure 2:**
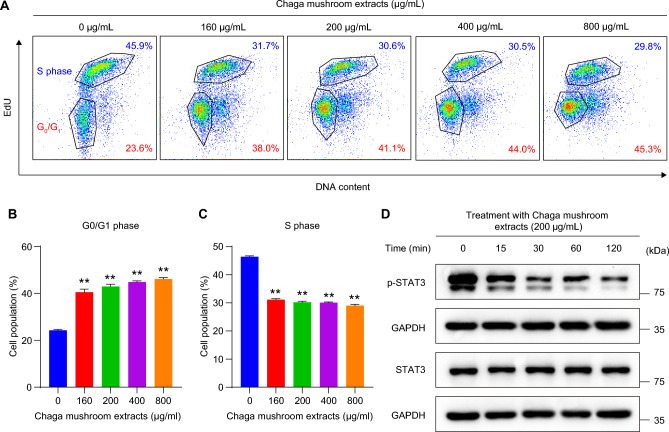
Chaga mushroom extract inhibits the cell cycle in oral cancer cells via downregulation of p-STAT3. (**A**) Flow cytometry analysis on the cell cycle in HSC-4 cells after treatment (0, 160, 200, 400, and 800 μg/mL). (**B**) Quantification of G0/G1 phase in HSC-4 cells after treatment. (**C**) Quantification of the S phase in HSC-4 cells after treatment (0, 160, 200, 400, and 800 μg/mL). (**D**) Expression level of phosphorylated STAT3 (p-STAT3) and total STAT3 (STAT3) in HSC-4 cells after treatment (200 μg/mL) for the indicated time points (0, 15, 30, 60, and 120 min). Values represent the mean ± SEM. **p < 0.01 vs. untreated HSC-4 cells.

To investigate the effect of Chaga mushroom extract on oral cancer cell behavior, such as viability, proliferation, cell cycle, glycolysis, mitochondrial respiration, and apoptosis, HSC-4 was treated with the extract at different concentrations (0, 160, 200, 400, and 800 µg/mL) for 24 h. The analysis results are shown in Fig. 1A. First, to determine whether Chaga mushroom extract controls the survival and proliferation of oral cancer cells, HSC-4 cells were treated with 0, 160, 200, 400, and 800 µg/mL of extract and cell viability and proliferation were assessed (Fig. 1B,C). The extract inhibited cell viability and proliferation in a dose-dependent manner (Fig. 1B,C). A cell cycle analysis was subsequently performed on the HSC-4 cells (Fig. 2A). Chaga mushroom extract significantly increased the G0/G1 phase in the HSC-4 cells compared with that in untreated HSC-4 cells (Fig. 2B). Additionally, the S phase was significantly decreased in the treated HSC-4 cells compared to that in the untreated cells (Fig. 2C). Accumulating evidence has shown that STAT3 signaling is crucial for tumor microenvironment and cancer development, including cell survival and proliferation^19^. To determine whether the inhibitory effects of Chaga mushroom extract on cancer survival and proliferation in HSC-4 cells involved STAT3 signaling, activation of STAT3 was assessed after treatment of the cells with the extract (200 µg/mL). Western blot analysis showed that the extract drastically decreased the expression of phospho-STAT3 (p-STAT3) within 15 min and decreased the level of p-STAT3, which was maintained for 120 min (Fig. 2D). These results indicate that Chaga mushroom extract is likely to decrease the cell cycle by suppressing STAT3 activation, leading to the inhibition of cell viability and proliferation in oral cancer cells. To further identify which components in Chaga mushroom extract are responsible for the anti-cancer effect, we conducted LC–MS/MS analysis on Chaga mushroom extract. In the LC–MS/MS analysis, we identified 16 candidate molecules (Supplementary Fig. S1A,B, Supplementary Tables S1 and S2). Among these candidates, we selected three potential anti-cancer molecules—syringic acid, protocatechuic acid, and 2-hydroxy-3,4-dimethoxybenzoic acid—based on prior literature^20–23^. To determine their concentrations in Chaga mushroom extract, we performed HPLC–DAD analysis. The results of HPLC–DAD assay revealed that the quantities of each molecule present in the Chaga mushroom extract (Supplementary Fig. S2A–C, Supplementary Table S3).

### Chaga mushroom extract regulates glycolysis in oral cancer cells

**Figure 3:**
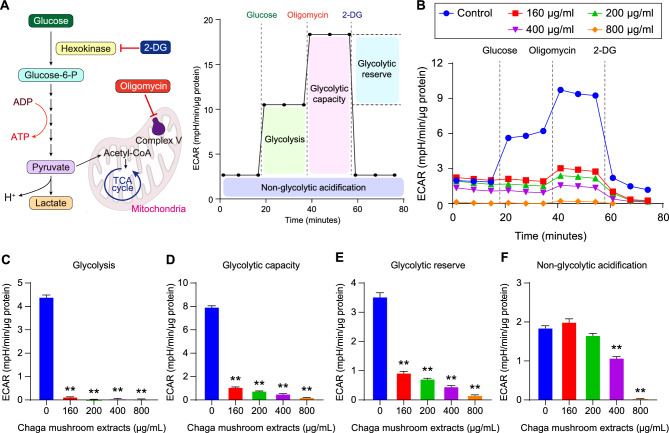
Chaga mushroom extract reduces glycolysis in oral cancer cells. (**A**) Schematic illustration of glycolysis and extracellular acidification rate (ECAR) measurement procedures. ECAR was measured following consecutive treatments with glycolysis-involved reagents such as glucose, oligomycin, and 2-deoxyglucose (2-DG). (**B**) Real time analysis of ECAR in HSC-4 cells after treatment (0, 160, 200, 400, and 800 μg/mL). (**C**–**F**) Glycolysis (**C**), glycolytic capacity (**D**), glycolytic reserve (**E**), and non-glycolytic acidification (**F**) in HSC-4 cells after treatment (0, 160, 200, 400, and 800 μg/mL). Values represent the mean ± SEM. **p < 0.01 vs. untreated HSC-4 cells.

Cancer cell metabolism is characterized by increased glucose uptake and glycolysis under aerobic conditions, a phenomenon known as the Warburg effect^24^. Thus, the regulation of glycolysis is a powerful strategy for destroying cancer cells. To examine whether Chaga mushroom extract regulates glycolysis in oral cancer cells, the extracellular acidification rate (ECAR) was measured using a Seahorse XF glycolysis stress assay (Fig. 3A). To assess glycolysis, glycolytic capacity, glycolytic reserves, and non-glycolytic acidification, ECAR measurements were performed in real-time in treated HSC-4 cells at different concentrations (0, 160, 200, 400, and 800 µg/mL) after injection of glucose, mitochondrial electron transport chain complex V inhibitor oligomycin, and hexokinase inhibitor 2-DG (Fig. 3A,B). Glycolysis, glycolytic capacity, and glycolytic reserves significantly decreased in the treated HSC-4 cells in a dose-dependent manner (Fig. 3C–E). Non-glycolytic acidification is involved in the TCA cycle CO2-derived acidification^25^. Intriguingly, Chaga mushroom extract at concentrations of 400 and 800 µg/mL affected cellular physiology other than glycolysis (Fig. 3F). These data indicate that the extract inhibits glycolysis process by suppressing glycolysis, glycolytic capacity, and glycolytic reserves.

### Chaga mushroom extract induces autophagy via AMPK activation

**Figure 4:**
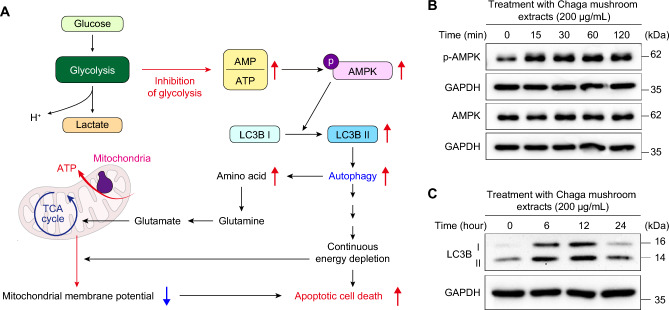
Chaga mushroom extract activates autophagy processes via the AMPK-LC3B-II axis. (**A**) Schematic representation of autophagy-mediated apoptotic cell death. (**B**) Expression levels of phosphorylated AMPK (p-AMPK) and total AMPK (AMPK) in HSC-4 cells after treatment (200 μg/mL) for the indicated time points (0, 15, 30, 60, and 120 min). (**C**) Expression levels of LC3B-I and II in HSC-4 cells after treatment (200 μg/mL) for the indicated time points (0, 6, 12, and 24 h).

Autophagy serves as a protective mechanism, preventing cellular energy generation while removing defective components under glucose-deprived conditions, whereas sustained autophagy under energy-depleted conditions triggers apoptotic cell death (Fig. 4A)^26^. Our findings have revealed that Chaga mushroom extract suppressed glycolysis in oral cancer cells (Fig. 3). Thus, to investigate whether it induces autophagy as well, we examined the activation of AMPK, a key energy sensor that maintains cellular energy metabolism^27^. Western blot analysis indicated that treatment with Chaga mushroom extract facilitated the phosphorylation of AMPK within 15 min and that the increased level of p-AMPK was maintained for 120 min (Fig. 4B). Additionally, the expression of LC3B-II was significantly enhanced after treatment with the extract for 24 h (Fig. 4C). Furthermore, inhibition of autophagy by treatment with chloroquine, which is a reagent for autophagy inhibitor, decreased apoptosis (Supplementary Fig. S3). These findings suggest that Chaga mushroom extract induces autophagy in oral cancer cells and may induce autophagy-mediated cell death by inhibiting glycolysis.

### Chaga mushroom extract decreases mitochondrial spare respiratory capacity through continuous glycolysis inhibition

When glycolysis is suppressed under glucose deprivation or pathophysiological conditions, mitochondrial respiration is an alternative mechanism for supplying energy to the cell. Under glucose-deprived conditions, mitochondria utilize amino acids as a source of substrates for mitochondrial respiration, which are generated by autophagy. However, under these conditions, continuous glucose depletion reduces mitochondrial membrane potential, resulting in autophagy-mediated cell death.

**Figure 5:**
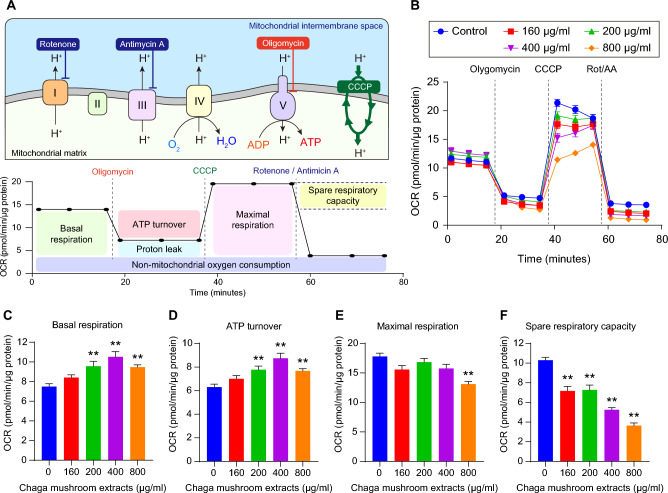
Chaga mushroom extract suppresses mitochondrial membrane potential in oral cancer cells. (**A**) Graphical illustration of mitochondrial electron transport chain and oxygen consumption rate (OCR) measurement procedures. OCR was analyzed following consecutive treatments with electron transport chain modulators such as oligomycin, CCCP, and a combination of rotenone and antimycin A. (**B**) Real-time analysis of OCR in HSC-4 cells after treatment (0, 160, 200, 400, and 800 μg/mL). (**C**–**F**) Mitochondrial basal respiration (**C**), ATP turnover (**D**), mitochondrial maximal respiration (**E**), and mitochondrial spare respiratory capacity (**F**) in HSC-4 cells after treatment (0, 160, 200, 400, and 800 μg/mL). Values represent the mean ± SEM. **p < 0.01 vs. untreated HSC-4 cells.

To examine whether Chaga mushroom extract is involved in mitochondrial respiration, the oxygen consumption rate (OCR) of oral cancer cells was measured using the Seahorse XF Mito stress test after treatment with the extract (Fig. 5A,B). To supply ATP to glycolysis-inhibited oral cancer cells treated with the extract, the induction of autophagy (Fig. 4) increased mitochondrial basal respiration and ATP turnover in a dose-dependent manner (Fig. 5C,D). However, with no change in maximal mitochondrial respiration—except for at the 800 µg/mL extract concentration—the mitochondrial spare respiratory capacity was significantly reduced in a dose-dependent manner (Fig. 5E,F). These results indicate that Chaga mushroom extract suppresses the mitochondrial membrane potential in oral cancer cells by continuous glycolysis inhibition-mediated autophagy, suggesting that mitochondrial dysfunction leads to apoptotic cell death. As shown in Fig. 3A, high concentrations (800 µg/mL) of this extract may affect other cellular physiologies, leading to a reduction in both mitochondrial maximal respiration and spare respiratory capacity.

### Chaga mushroom extract is likely to increase apoptosis through p38 MAPK and NF-κB activation

**Figure 6:**
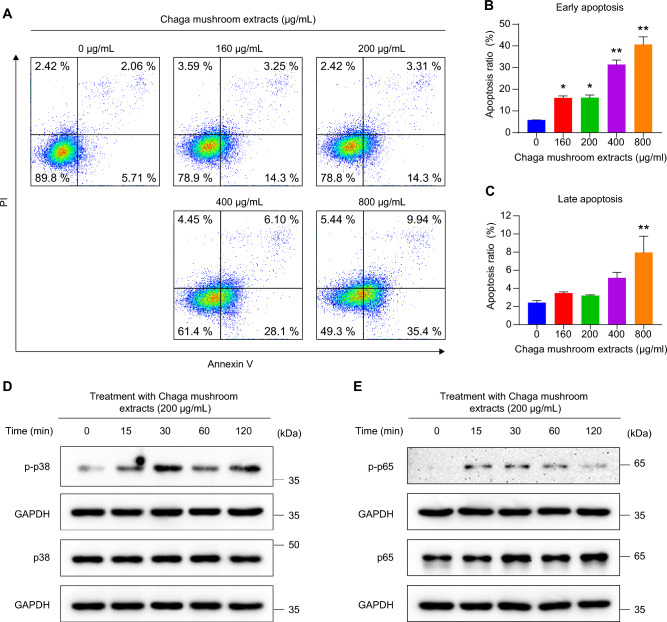
Chaga mushroom extract induces apoptosis of HSC-4 cells through activation of p38 MAPK and NF-κB. (**A**) Apoptosis assay of HSC-4 cells after treatment (0, 160, 200, 400, and 800 μg/mL). (**B**) Quantification of early apoptotic ratio of HSC-4 cells after treatment (0, 160, 200, 400, and 800 μg/mL). (**C**) Quantification of late apoptotic ratio of HSC-4 cells after treatment (0, 160, 200, 400, and 800 μg/mL). (**D**) Expression levels of phosphorylated p38 (p-p38) and total p38 (p38) in HSC-4 cells after treatment (200 μg/mL) for the indicated time points (0, 15, 30, 60, and 120 min). (**E**) Expression levels of phosphorylated p65 (p-p65) and total p38 (p65) in HSC-4 cells after treatment (200 μg/mL) for the indicated time points (0, 15, 30, 60, and 120 min). Values represent the mean ± SEM. *p < 0.05; **p < 0.01 vs. untreated HSC-4 cells.

Our findings have shown that Chaga mushroom extract inhibits energy metabolism processes, including glycolysis and mitochondrial membrane potential, leading to autophagy (Figs. 3, 4, 5). To verify whether Chaga mushroom extract-mediated continuous energy depletion induces autophagy-associated apoptotic cell death in oral cancer cells, flow cytometric analysis of Annexin V and propidium iodide in these cells was performed after treatment with the extract for 6 h (Fig. 6A). Chaga mushroom extract increased early apoptosis of oral cancer cells in a dose-dependent manner (Fig. 6B); however, late apoptosis did not show a significant difference in the range of 0–400 µg/mL extract concentration (Fig. 6C). Activated p38 MAPK induces apoptosis through several stressors, including oxidative and environmental stress and chemical reagents^28,29^. Additionally, stimulation of the NF-κB pathway promotes apoptosis in response to cellular stress and inflammatory responses^30^. To establish whether Chaga mushroom extract induces p38 MAPK- and NF-κB-stimulated apoptosis, the activation of p38 and p65—complexes of NF-κB—was assessed in HSC-4 cells after treatment with the extract (200 µg/mL). Western blot analysis showed that the extract increased the levels of p-p38 and p-p65 within 15 min (Fig. 6D,E), indicating that Chaga mushroom extract is likely to induce apoptosis via activation of p38 MAPK and NF-κB pathways.

## Discussion

Several studies have shown the antitumor effects of Chaga mushrooms against various cancers, including lung adenocarcinoma, breast cancer, liver carcinoma, and colorectal cancer^6,7,10,31^. Chaga mushroom extract inhibits the growth of breast cancer cells by inducing autophagy processes^6^. Chaga mushroom constituents exhibit cytotoxic and proapoptotic activities in human lung cancer cells^7^. In addition, Chaga mushroom-derived ergosterol peroxide suppresses proliferation of colorectal cancer cells through down-regulation of β-catenin signaling^10^. Similarly, we demonstrated that Chaga mushroom extract decreases the proliferation of oral cancer cells by modulating cancer energy metabolism and autophagy. Furthermore, we have shown the anti-tumor effect of this extract in oral cancer cells through regulation of several cell signal pathways, including AMPK, STAT3, p38 MAPK, and NF-κB. This study, for the first time, found that Chaga mushroom extract suppresses oral cancer cell progression by inhibiting glycolysis and mitochondrial membrane potential.

STAT3 is a key regulator of cancer progression^32,33^. The STAT3 signaling pathway participates in cell proliferation in several types of cancers, such as liver, breast, colorectal, gastric, and lung cancers^34^. Moreover, phosphorylation of STAT3 signaling is involved in several cancer physiologies, including apoptosis, glycolysis, and epithelial-mesenchymal transition (EMT). In hepatocellular carcinoma, the inhibition of the JAK1-STAT3 pathway decreases cancer cell progression by suppressing cell growth^35^. Silencing STAT3 expression inhibits cell growth in colorectal cancer cells through G2 phase arrest, inducing apoptosis^36^. Furthermore, the activation of STAT3 signaling increases glycolysis, leading to an increase in the growth rate of prostate cancer cells^37^. Moreover, STAT3 signaling promotes EMT in colorectal cancer, hepatocellular carcinoma, and prostate cancer^37–39^. These findings indicate that the inhibition of STAT3 might be a powerful strategy for the treatment of several cancers. Our results indicated that Chaga mushroom extract was likely to decrease the level of p-STAT3 in oral cancer cells, thereby suppressing cell growth and glycolysis by inhibiting the STAT3 signaling pathway.

Since the Warburg effect was initially described, it has been shown to be the main characteristic of cancer progression. Fluorodeoxyglucose positron emission tomography has shown that most primary and metastatic human cancers exhibit enhanced glucose uptake, resulting in a high glycolytic rate^40^. Thus, glycolysis attenuation is essential for preventing the proliferation, invasion, metastasis, and development of cancer^41^. Glycolysis inhibition reduces the generation of ATP, which promotes the activation of the AMPK signaling pathway, thereby inducing autophagy to compensate for insufficient energy. Generally, autophagy is a cytoprotective physiological process that prevents cellular stress such as energy depletion and the removal of dysfunctional components; however, continuous autophagic flux leads to autophagy-dependent cell death^26^. This study verified that Chaga mushroom extract inhibited glycolysis in oral cancer cells and induced autophagy by upregulating p-AMPK and LC3B-II levels, resulting in apoptosis induction. Specifically, our data showed that this extract decreased mitochondrial spare respiratory capacity, indicating that it reduced mitochondrial membrane potential. Because mitochondrial electron transport chain activity is an alternative process when there is insufficient ATP under glycolysis-inhibited conditions, a decreased mitochondrial membrane potential induces a sustained autophagy process, resulting in cell death. Our results indicate, for the first time, that Chaga mushroom extract facilitates autophagy-dependent apoptosis in oral cancer cells by suppressing glycolysis and mitochondrial membrane potential.

Our data revealed that treatment with Chaga mushroom extract increased the levels of p-p38 and p-p65 in oral cancer cells. p38—a proline-directed serine/threonine kinase of the MAPK family—is activated by various environmental stressors such as oxidative and osmotic stress, UV or gamma radiation, and several cytokines and inflammatory molecules, contributing to various biological and pathophysiological responses^29^. The inactivation of p38 leads to stemness of cancer stem cells in non-small cell lung cancer^42^. In addition, phosphorylation of p38 decreases the level of epidermal growth factor receptor expression, resulting in the attenuation of the sphere-forming capacity and self-renewal of glioma cancer stem cells^43^. As a transcription factor, NF-κB is involved in immune responses, and in several types of cancers it can positively and negatively affect tumor development and progression^44^. In certain circumstances, both activation of NF-κB and STAT3 promote pro-tumorigenic effects, including augmentation of cancer proliferation and anti-apoptotic gene expression^44^. However, our results demonstrated that Chaga mushroom extract decreased the expression of p-STAT3 and increased the expression of p-p65. Since STAT3 inhibits NF-κB-mediated anti-tumor immunity^45^, a previous study explored whether the activation of NF-κB suppresses tumor progression in *STAT3*-silencing hepatocellular carcinoma^46^. Additionally, one study showed that the activation of p38 MAPK and NF-κB inhibits cancer stem cell-like properties and migratory capacity in malignant human keratinocytes^47^. These findings indicate that Chaga mushroom extract promotes apoptosis through activation of p38 MAPK and NF-κB signal pathways.

Through the LC–MS/MS analysis and HPLC–DAD assay, our findings provided potential anti-cancer components from Chaga mushroom extract and identified the quantities of three anti-cancer molecules in Chaga mushroom extract, such as syringic acid, protocatechuic acid, and 2-hydroxy-3,4-dimethoxybenzoic acid. However, we recognize limitations in this study. Firstly, although Chaga mushroom extract induced apoptotic cell death in flow cytometry analysis upon staining with Annexin V and PI (Fig. 6A–C), the expression of cleaved caspase-3 did not detect (Data not shown). However, the expression of pro-apoptotic protein, Bax was increased in HSC-4 cells after treatment with Chaga mushroom extract (Supplementary Fig. S4). This phenomenon might represent caspase-independent apoptosis^48,49^. Caspase-independent apoptosis is induced by by Bcl2/adenovirus E1B 19kDa interacting protein 3 (BINP-3), which activates the release of mitochondrial AIF, leading to cell death through chromatin condensation, DNA damage, ROS production, and proteolysis^50^. Since there are several anti-cancer components in Chaga mushroom extract, further study is needed to investigate how each potential anti-cancer molecule induces caspase-independent apoptosis in oral cancer cells. Secondly, we confirmed that Chaga mushroom extract leads to autophagy-mediated cell death (Fig. 4, Supplementary Fig. S3). However, regarding cell signaling on p38 MAPK and NF-kB pathway, it is necessary to determine whether these pathways are involved in Chaga mushroom extract-induced cell death. Activation of p38 MAPK and NF-kB pathway generally induces pro-apoptotic processes^30,51^, and our data have shown that the expression of p-p38 and p-p65 were increased after treatment with Chaga mushroom extract in a time dependent manner (Fig. 6D,E). These findings indicate that p38 MAPK and NF-kB could be involved in Chaga mushroom extract-mediated cell death, but we could not perform the inhibition assay of these pathways because Chaga mushroom extract used in this study consists of multiple anti-cancer component, and it is unclear which specific component might regulate these pathways. Thus, further study is needed to elucidate the precise mechanism of the three potential anti-cancer reagents in oral cancer cell by regulating p38 MAPK and NF-kB pathways, as well as caspase-independent pathway.

## Conclusions

**Figure 7:**
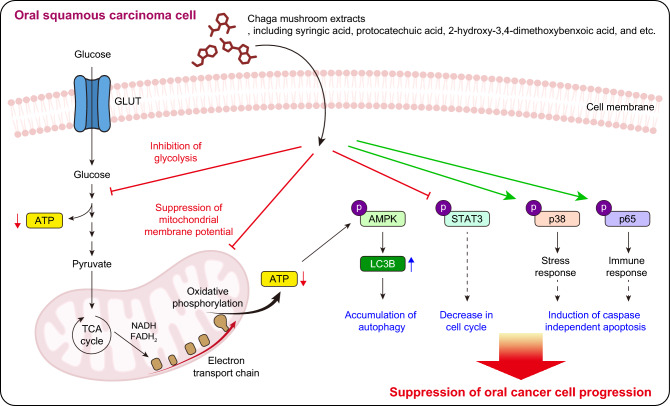
Schematic representation of proposed mechanisms by which Chaga mushroom extract suppresses oral cancer cell progression. It decreases glycolysis and mitochondrial membrane potential, leading to accumulation of autophagy through activation of AMPK signal axis. In addition, it inhibits the cell cycle via suppression of p-STAT3. Consequently, it induces apoptosis of oral cancer cells by activating p38 MAPK and NF-κB, resulting in suppression of oral cancer cell progression.

Collectively, these results demonstrate that Chaga mushroom extract inhibits glycolysis and mitochondrial membrane potential in oral cancer cells, resulting in reduced ATP levels. Consequently, autophagy is induced by AMPK activation, and the cell cycle is decreased by dephosphorylation of STAT3, leading to apoptosis of oral cancer cells through the activation of p38 MAPK and NF-κB (Fig. 7). While further pre-clinical studies are warranted to assess whether the administration of Chaga mushroom extract inhibits tumor progression, our results suggest its viability as a supplementary therapeutic agent for treating patients with oral cancer.

## Methods

### Cell culture and materials

The human oral cancer cell line (HSC-4) was obtained from the Korea Cell Line Bank (Seoul, Republic of Korea). The cells were maintained in DMEM supplemented with 10% fetal bovine serum and 100 U/ml penicillin–streptomycin (Thermo Fisher Scientific, Waltham, MA, USA) at 37 °C with 5% CO_2_ in a humidified incubator. Chaga mushroom extract was purchased from HUMANAR. Co., Ltd. (Asan, Republic of Korea). Chaga mushroom extract was dissolved in the distilled water.

### Cell viability assay

A cell counting kit-8 (CCK-8) assay (Dojindo Laboratories, Kumamoto, Japan) was used to measure the viability of HSC-4 cells after treatment with Chaga mushrooms, according to the manufacturer’s instructions. Briefly, exponentially growing HSC-4 cells were seeded in a 96-well plate at a concentration of 7,000 cells per well and treated with Chaga mushroom extract at concentrations of 160, 200, 400, and 800 µg/mL for 24 h. After incubation, 10% CCK-8 reagent was added to each well and the plate was incubated for 1 h. Absorbance was measured at 450 nm using a Varioskan LUX Multimode microplate reader (Thermo Fisher Scientific).

### Cell proliferation assay

Cell proliferation of HSC-4 cells was analyzed using a BrdU cell proliferation assay kit (Cell Signaling Technology, Danvers, MA, USA). Briefly, equal numbers of HSC-4 cells were seeded in a 96-well plate at a density of 7000 cells per well and treated with Chaga mushroom extract at concentrations of 160, 200, 400, and 800 µg/mL for 24 h. The BrdU assay was performed after treatment with BrdU for 24 h. The absorbance was measured at 450 nm using a Varioskan LUX Multimode microplate reader (Thermo Fisher Scientific).

### Flow cytometry analysis

For cell cycle analysis, Chaga mushroom-treated cells were labelled with EdU (Thermo Fisher Scientific) for 2 h before harvesting. Cells were collected and stained with the Click-iT Plus EdU Alexa Fluor 488 flow cytometry kit (Thermo Fisher Scientific) following the manufacturer’s instructions. After washing, DNA was stained with FxCycle™ Far Red stain (Thermo Fisher Scientific) according to the manufacturer’s protocol. The cells were then acquired on a BD Accury C6 Plus (BD Bioscience, Franklin Lakes, NJ, USA), followed by analysis using FlowJo software (BD Bioscience). For apoptosis analysis, the cells were seeded at a density of 5 × 10^5^ cells in a 60 mm culture dish. After 24 h, the cells were treated with various concentrations of Chaga mushroom extract (0, 160, 200, 400, and 800 µg/mL) for 6 h, harvested by trypsinization, and collected by centrifugation. The cells were washed with cold PBS, resuspended in 100 µl Annexin V binding buffer, and stained with 5 µl FITC-conjugated Annexin V and 5 µl propidium iodide in the dark for 20 min at room temperature. After incubation, 400 µl binding buffer was added and apoptotic cells were analyzed according to the manufacturer’s instructions (Annexin V-FITC apoptosis detection kit, BD Bioscience) using a BD Accury C6 plus flow cytometer (BD Bioscience). Cell distribution was analyzed using FlowJo software (BD Biosciences).

### Western blot analysis

Total cellular proteins were extracted by utilizing RIPA lysis buffer (Thermo Fisher Scientific). Cell lysates were separated by sodium dodecyl sulfate–polyacrylamide gel electrophoresis and proteins transferred onto nitrocellulose membranes (Bio-Rad Laboratories, Hercules, CA, USA). These membranes were blocked with 5% bovine serum albumin (Geneall, Seoul, Republic of Korea) and incubated with primary antibodies against STAT3, p-STAT3, AMPK, p-AMPK, LC3B, p38, p-p38, p65, p-p65, and glyceraldehyde 3-phosphate dehydrogenase (GAPDH) (Cell Signaling Technology). After primary antibody incubation, the membranes were incubated with horseradish peroxidase-conjugated goat anti-rabbit or anti-mouse IgG secondary antibodies (Cell Signaling Technology). The protein bands were visualized using an enhanced chemiluminescent substrate (Thermo Fisher Scientific).

### Assessment of glycolysis extracellular acidification rate

To assess glycolysis in oral cancer cells, the extracellular acidification rate (ECAR) was measured using an XFe96 extracellular flux analyzer (Agilent Technologies, Santa Clara, CA, USA) following the manufacturer’s instructions. Briefly, the HSC-4 cells were seeded at a density of 2 × 10^4^ cells/well in Seahorse XFe96 cell culture microplates (Agilent Technologies). After adherence and equilibration, Chaga mushroom extract was added at various concentrations (160, 200, 400, and 800 µg/mL) for 24 h. Then, the ECAR was measured through sequential injections of glucose (Sigma-Aldrich, Burlington, MA, USA), oligomycin (Sigma-Aldrich), and 2-deoxy glucose (2-DG; Sigma-Aldrich) following incubation with XF assay medium (Agilent Technologies). All ECAR data were normalized to the concentration of cellular protein per well using a Pierce™ BCA protein assay (Thermo Fisher Scientific).

### Assessment of mitochondrial respiration oxygen consumption rate

To analyze mitochondrial respiration, the OCR was assessed using an XFe96 extracellular flux analyzer (Agilent Technologies). The HSC-4 cells were seeded at a density of 2 × 10^4^ cells/well in Seahorse XFe96 cell culture microplates (Agilent, Santa Clara, CA, USA). After adherence and equilibration, the Chaga mushroom extract was treated with various concentrations (160, 200, 400, and 800 µg/mL) for 24 h. The OCR was then assessed by sequential injections of oligomycin (Sigma-Aldrich), carbonyl cyanide-p-trifluoromethoxyphenylhydrazone (CCCP; Sigma-Aldrich), rotenone (Sigma-Aldrich), and antimycin A (Sigma-Aldrich), followed by incubation with XF assay medium (Agilent Technologies). All OCR results were normalized to concentrations of cellular protein per well using a Pierce™ BCA protein assay (Thermo Fisher Scientific).

### Statistical analysis

All data are presented as mean ± standard error of the mean (SEM). The statistical significance of the results was determined using a one-way analysis of variance. Statistical significance was set at *p* < 0.05.

### Supplementary Information

Supplementary Figures.

Supplementary Tables.

## Data Availability

The datasets used and/or analyzed in the current study are available from the corresponding author upon reasonable request.
