# Supplementary material for: Chaga mushroom extract suppresses oral cancer cell growth via inhibition of energy metabolism
Source: Sci Rep. 2024 May 9;14:10616. doi: 10.1038/s41598-024-61125-z (PMC11078932; doi:10.1038/s41598-024-61125-z)

Supplementary Figure 1

A

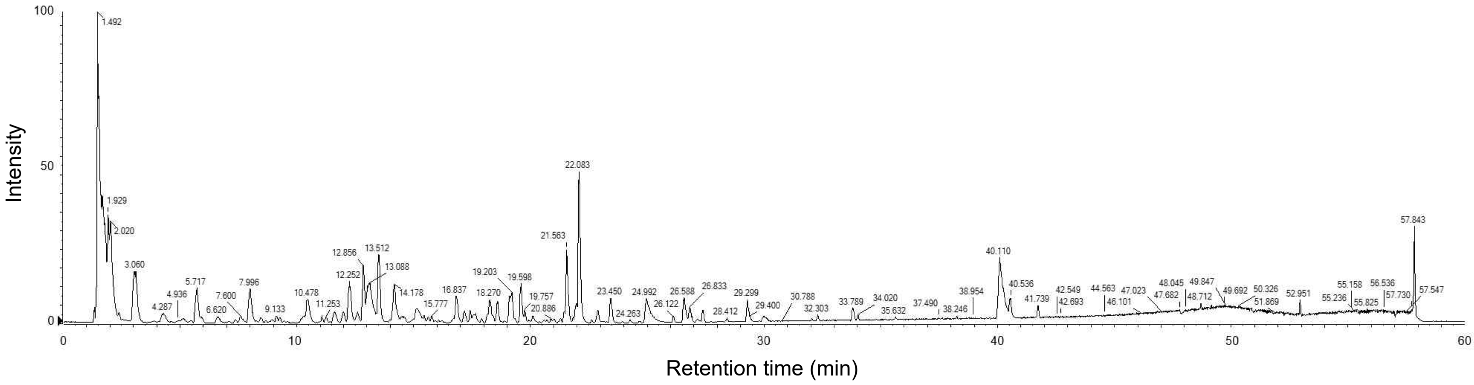

B

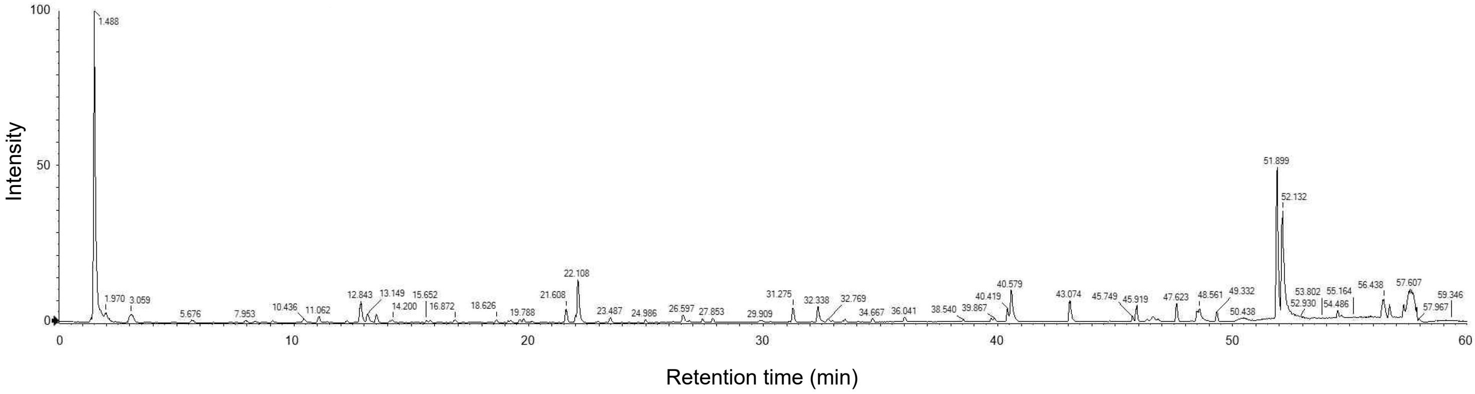

Supplementary Figure 2

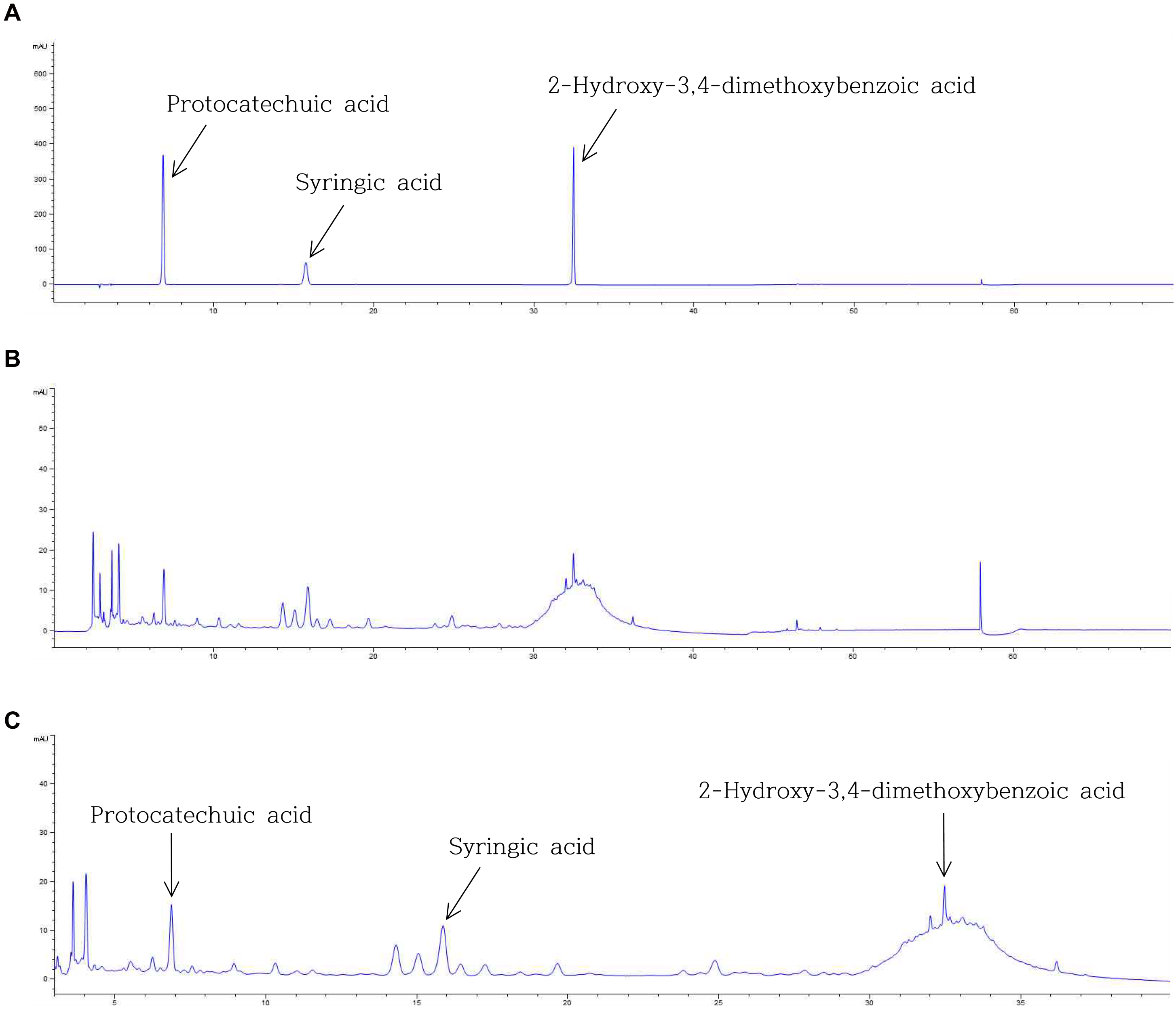

Supplementary Figure 3

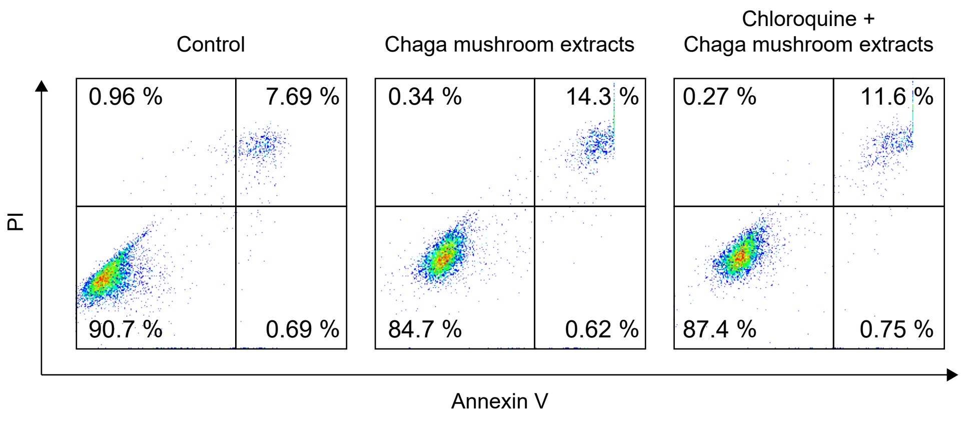

Supplementary Figure 4

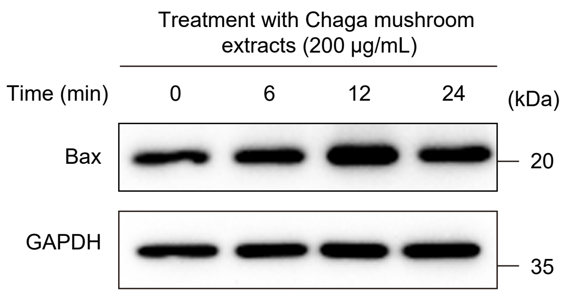

Supplementary Figure 5

Figure 2D p-STAT3

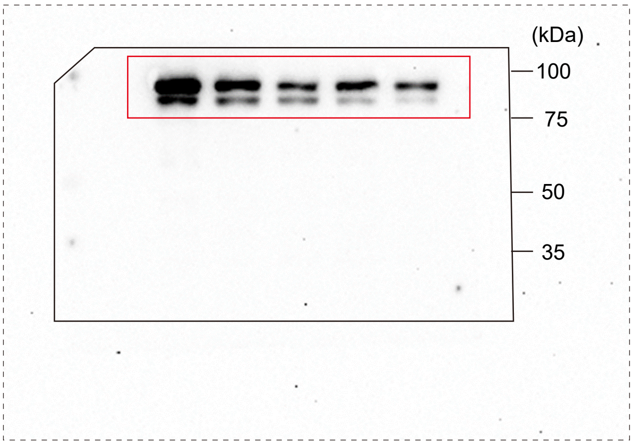

Figure 2D GAPDH for p-STAT3

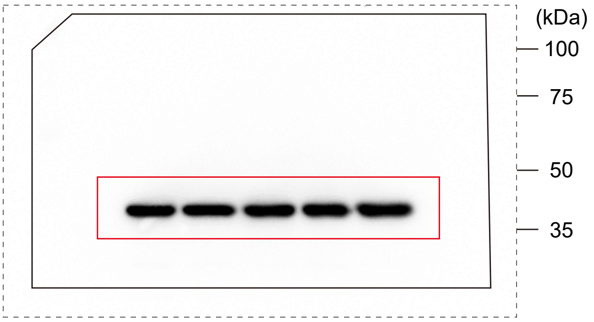

Figure 2D STAT3

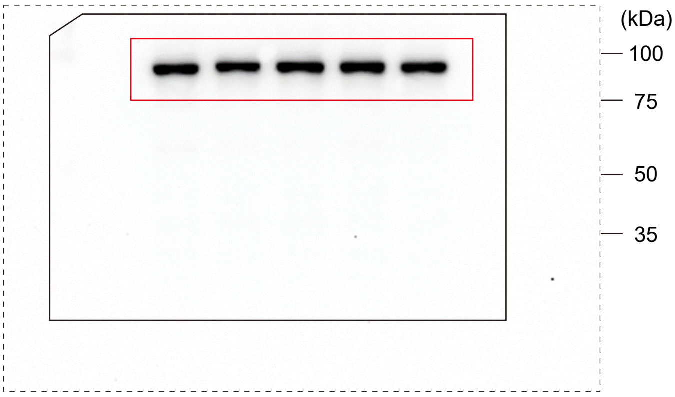

Figure 2D GAPDH for STAT3

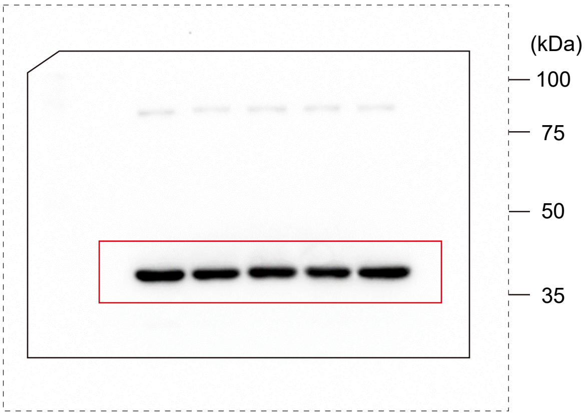

Figure 4B p-AMPK

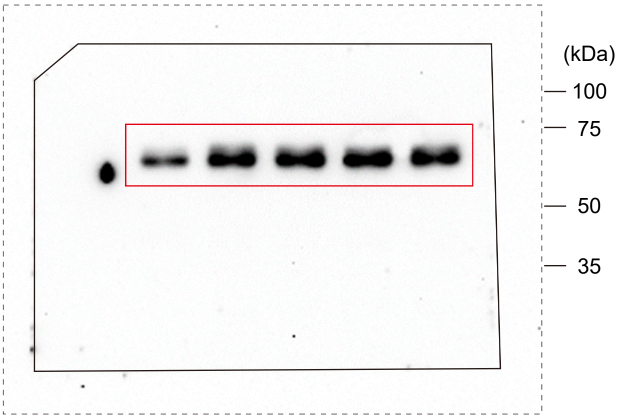

Figure 4B GAPDH for p-AMPK

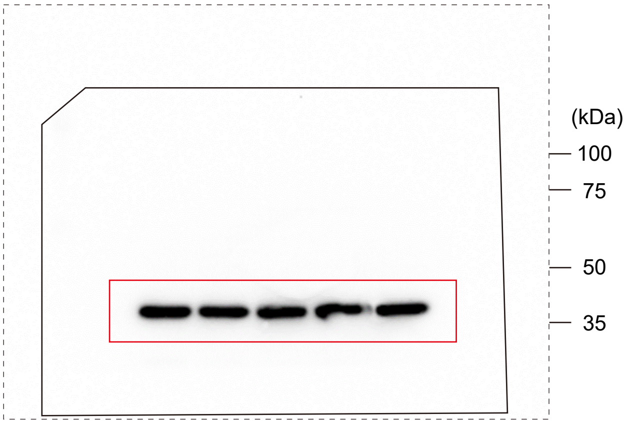

Figure 4B AMPK

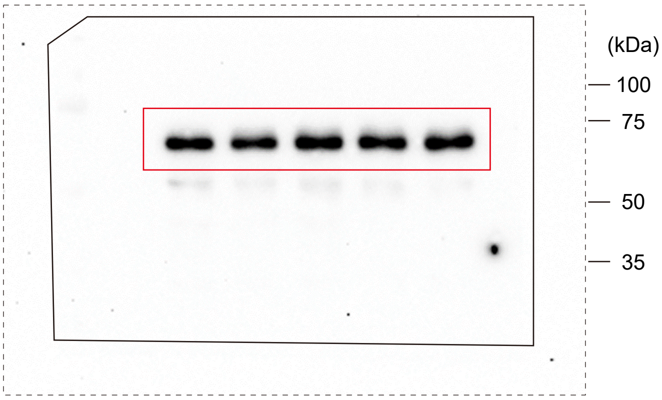

Figure 4B GAPDH for AMPK

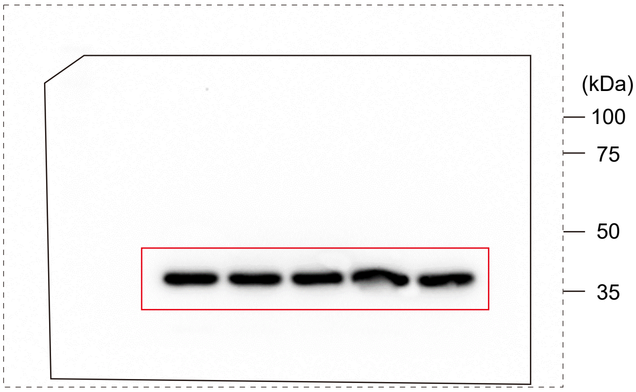

Supplementary Figure 5 (continued)

Figure 4C LC3B I/II
(Short time exposure)

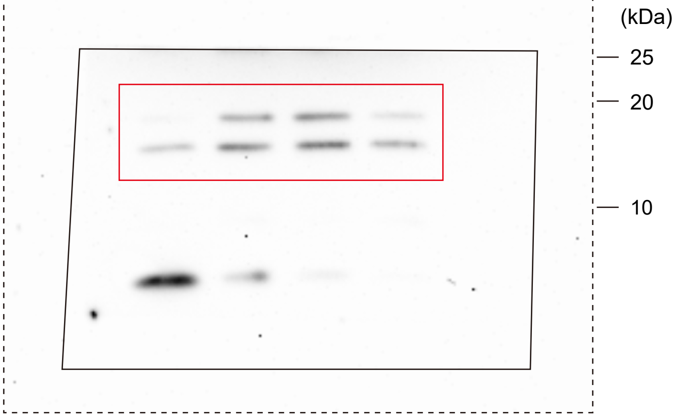

Figure 4C GAPDH for LC3B I/II

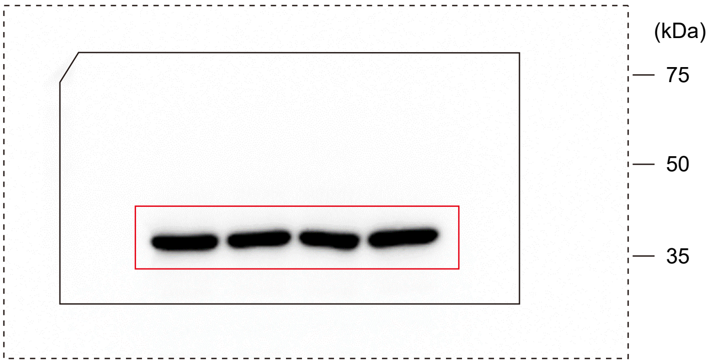

(Long time exposure)

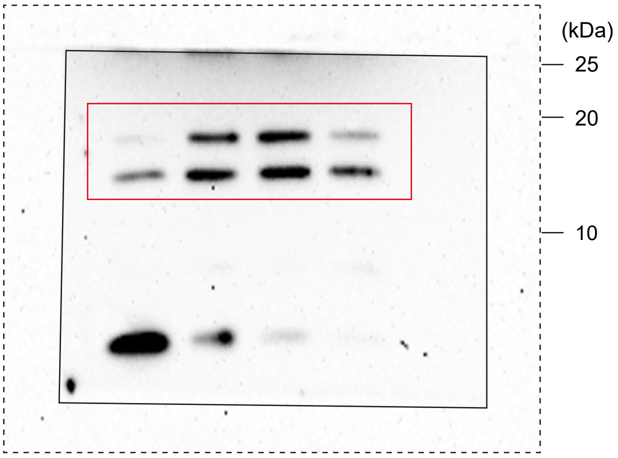

Figure 6D p-p38

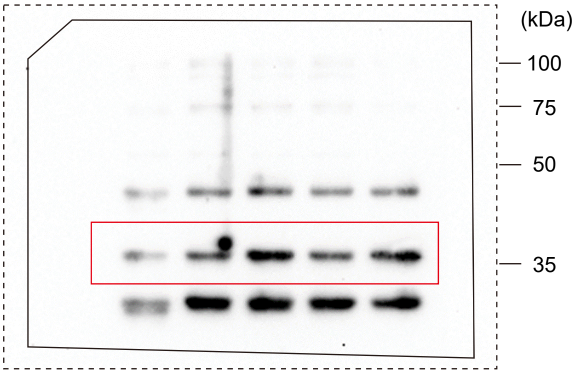

Figure 6D GAPDH for p-p38

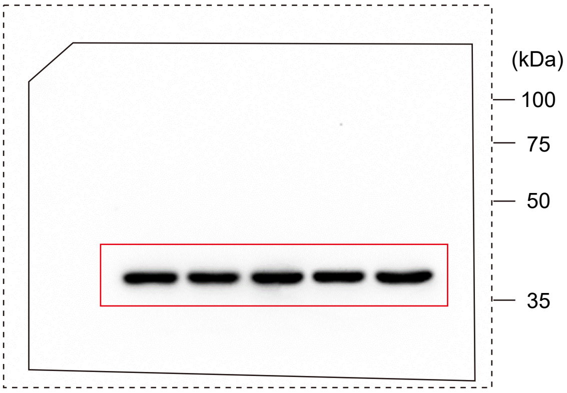

Figure 6D p38

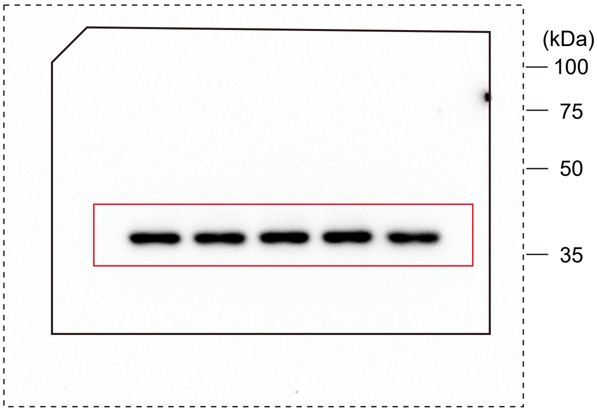

Figure 6D GAPDH for p38

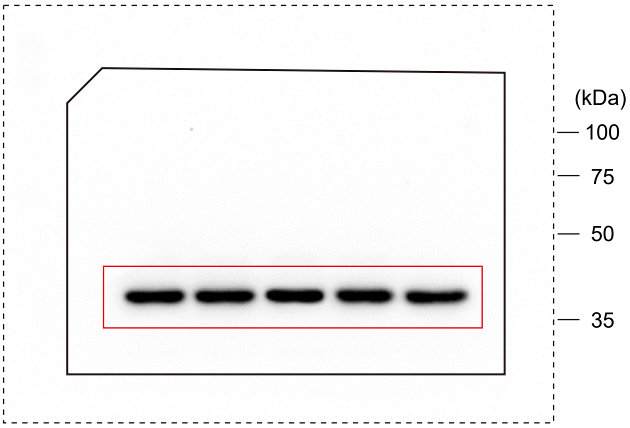

Supplementary Figure 5 (continued)

Figure 6E p-p65

(Short time exposure)

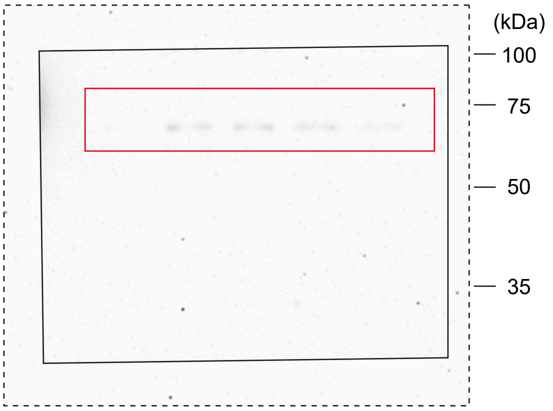

(Long time exposure)

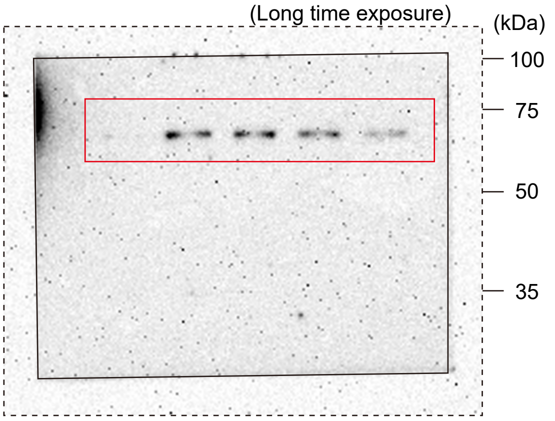

Figure 6E GAPDH for p-p65

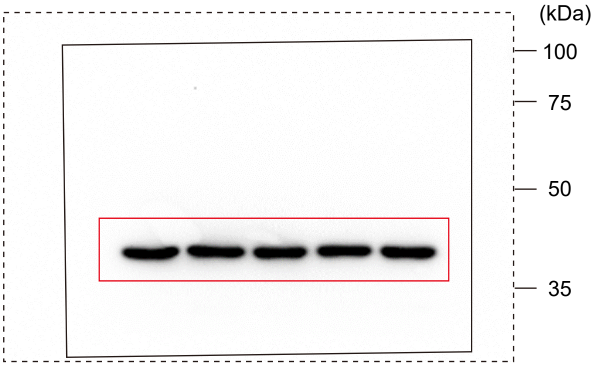

Figure 6E p65

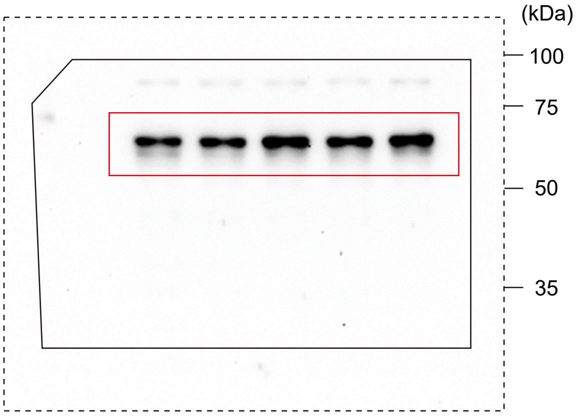

Figure 6E GAPDH for p65

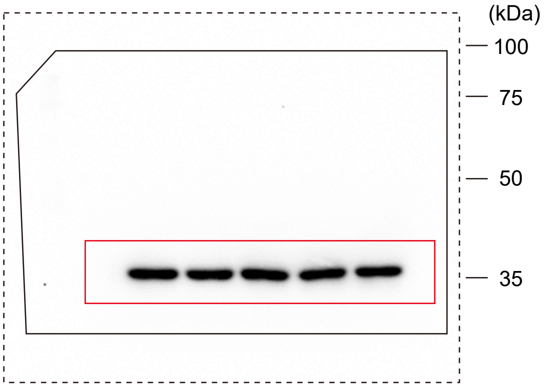

Supplementary Figure 4 Bax

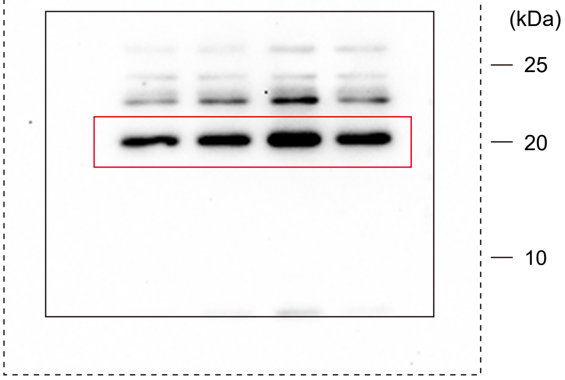

Supplementary Figure 4 GAPDH for Bax

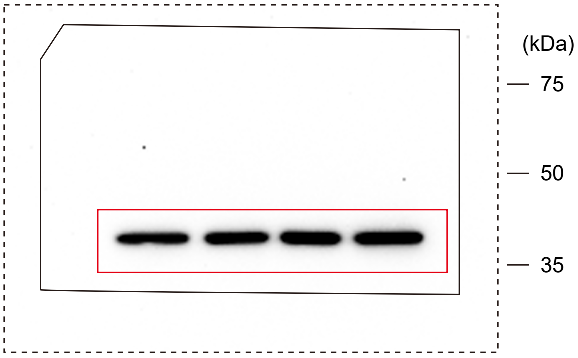

Supplement: Supplementary file 1 — Supplementary Figures. [file 41598_2024_61125_MOESM1_ESM.pdf]
