# Supplementary material for: Chaga mushroom extract suppresses oral cancer cell growth via inhibition of energy metabolism
Source: Sci Rep. 2024 May 9;14:10616. doi: 10.1038/s41598-024-61125-z (PMC11078932; doi:10.1038/s41598-024-61125-z)
Supplement: Supplementary file 2 — Supplementary Tables. [file 41598_2024_61125_MOESM2_ESM.docx]

**Supplementary Information**

**Supplementary Methods**

**LC-MS analysis**

Analysis was conducted using an LC system consisting of a Thermo Vanquish UHPLC with a Waters Cortex T3 column (150 mm × 2.1 mm, particle size 1.6 μm) maintained at 45 °C. The mobile phase was composed of water (eluent A: 0.1% HCOOH) and acetonitrile (eluent B: 0.1% HCOOH). The gradient was used, and the flow rate was set at 0.25 mL/min. Mass spectrometry was conducted on a Triple TOF 5600+ System (AB SCIEX, USA) equipped with a heated electrospray ion source. The mass spectrometer was operated in positive ion mode. Survey full-scan MS spectra (*m/z* 100–1500) were acquired using a quadrupole system with a resolution setting of 70,000. The spray voltage was set to 3.5 and 3.0 kV for the positive and negative ion modes, respectively. The top ten most intense precursor ions were selected for MS2 fragmentation, and spectra acquisition was performed using a resolution setting of 17,500. The remaining MS parameters were as follows: capillary temperature, 320 °C; sheath gas, 50 AU; sweep gas, 1 AU; auxiliary gas, 10 AU.

**HPLC-DAD analysis and calibration curves**

Chaga mushroom extract was dissolved in 80% MeOH and filtered through an 0.22 μm PVDF membrane filter (88.24 mg/mL). Chemical compounds were analyzed using HPLC (Agilent 1260 Infinity II Quat Pump, Santa Clara, CA, USA) with an INNO C18 column (4.6 × 250 mm, 5 μm). The mobile phase used water (0.1% trifluoroacetic acid) and acetonitrile, and the flow rate and sample injection volume were 1.0 mL/min and 10 μL, respectively. The gradient system was used, and analysis was monitored at 205 and 260 nm. Standard stock solutions (0.5 mg/mL) of each of compounds were prepared in 80% methanol, and repeatedly diluted with the same solvent. Calibration curves were prepared by plotting the concentrations of the standard solution with their respective peak areas. The linearity of the calibration curves was determined based on the correlation coefficient (*r^2^*), and the concentrations of reference compounds in the samples were then calculated from the calibration curve. The calibration functions were determined based on the peak area (*Y*), concentration (*X*, μg/mL), and mean ± standard deviation (*n* = 3).

**Supplementary Figure Legends**

**Supplementary Figure 1**. LC-MS/MS analysis of Chaga mushroom extract. **A** Proposed structures by LC-MS/MS analysis (negative mode). **B** Proposed structures by LC-MS/MS analysis (positive mode).

**Supplementary Figure 2**. HPLC-DAD analysis of Chaga mushroom extract. **A** HPLC-DAD chromatogram of standards. **B** HPLC-DAD chromatogram of Chaga mushroom extract sample. **C** Magnified HPLC-DAD chromatogram from (**B**).

**Supplementary Figure 3**. Autophagy-mediated HSC-4 cell death after treatment with Chaga mushroom extract. After pretreatment with chloroquine, autophagy inhibitor, apoptosis in HSC-4 cells treated with Chaga mushroom extract was assessed by flow cytometry analysis upon staining with Annexin V and PI.

**Supplementary Figure 4**. Expression of pro-apoptotic protein, Bax, after treatment with Chaga mushroom extracts.

**Supplementary Figure 5**. Uncropped whole membrane blot for western blot assay in this study. Western blot assay was performed on one membrane per one experimental set. Thus, empty lanes were cut, and bands were detected by reacting with antibodies. To enhance band detection, some blots were exposed for extended periods due to weak signal intensity (see Figure 4C for LC3B I/II and Figure 6E for p-p65). Moreover, when the target band size was under 25 kDa, membranes were cut to detect both the target bands and housekeeping bands (GAPDH; 36 kDa).

**Supplementary Table 1.** Proposed structures by LC-MS/MS analysis (negative mode)

| No. | Retention  time (min) | Molecular  weight | Tentative identification |
| --- | --- | --- | --- |
| 1 | 3.11 | 154.0 | Gentisic acid |
| 2 | 5.69 | 110.0 | Catechol |
| 3 | 5.72 | 154.0 | Protocatechuic acid |
| 4 | 5.82 | 210.0 | 1,2,4-Benzenetricarboxylic acid |
| 5 | 7.62 | 184.0 | 4-O-Methylgallic acid |
| 6 | 7.99 | 138.0 | Protocatechuic aldehyde |
| 7 | 10.52 | 152.0 | 3,4-Dihydroxyacetophenone |
| 8 | 11.07 | 168.0 | 5-Methoxysalicylic acid |
| 9 | 12.25 | 182.0 | 5-Hydroxyisophthalic acid |
| 10 | 12.60 | 166.0 | 1,4-Benzenedicarboxylic acid |
| 11 | 12.67 | 236.0 | 6,7-Dihydroxycoumarin-4-acetic acid |
| 12 | 12.85 | 198.1 | Syringic acid |
| 13 | 13.12 | 226.0 | 2,4,5-Phenoltricarboxylic acid |
| 14 | 13.13 | 236.0 | 6,7-Dihydroxycoumarin-4-acetic acid |
| 15 | 13.51 | 198.1 | 2-Hydroxy-3,4-Dimethoxybenzoic acid |
| 16 | 15.13 | 210.0 | 1,2,4-Benzenetricarboxylic acid |

**Supplementary Table 2.** Proposed structures by LC-MS/MS analysis (Positive mode)

| No. | Retention  time (min) | Molecular  Weight | Tentative identification |
| --- | --- | --- | --- |
| 1 | 12.85 | 198.1 | Syringic acid |
| 2 | 13.13 | 236.0 | 6,7-Dihydroxycoumarin-4-acetic acid |

**Supplementary Table 3.** Assessment of anticancer components in Chaga mushroom extract by HPLC-DAD analysis

| Components | Syringic acid | Protocatechuic acid | 2-hydroxy-3,4-dimethoxybenzoic acid |
| --- | --- | --- | --- |
| Amount in Chaga mushroom extract  (μg/g) | 160.64 ± 0.92 | 72.87 ± 0.52 | 14.19 ± 0.32 |
